# Surgical fixation with K-wires versus casting in adults with fracture of distal radius: DRAFFT2 multicentre randomised clinical trial

**DOI:** 10.1136/bmj-2021-068041

**Published:** 2022-01-19

**Authors:** Matthew L Costa, Juul Achten, Alexander Ooms, May Ee Png, Jonathan A Cook, Sarah E Lamb, Helen Hedley, Joseph Dias, DRAFFT2 Collaborators, Melina Dritsaki, Helen Dakin, Jonathan Jones, Andrew Mckee, Kevin Smith, Mohamed Hamadto, Steve Gwilym, Tim Chesser, Jaime Candal-Couto, Caroline Hing, David Giddin, Phil Johnston, Aamer Ullah, John Williams, Will Eardley, Makaram Srinivasan, Sridharrao Sampalli, Mark Farrar, Chris Roberts, Khitish Mohanty, Iain MacLeod, Praveen Sarda, Amr Elseehy, Nigel Rossiter, David Warwick, Chris Peach, David MacKay, Richard Benson, Adam Watts, Jonathan Young, Feisal Shah, Stephen Lipscombe, Aaron Ng, Charalambos P Charalambous, Barnaby Sheriden, Kanthan Theivendran, Pulimamidi Sanjay, Rajesh Nanda, Antony Bateman, Michael Butler, Oliver Keast-Butler, Andrew McAndrew, Wystan Chevannes, Pradeep Kankanalu, Asanka Wijendra, Andreas Fontalis, Hytham Afifi, Marie-Clare Killen, Ryan Higgin, Warran Wignadasan, Ken Wong, Catherine Gibson, Harry Beale, Bob Jennings, James Kennedy, Mark Williamson, Damir Rasidovic, Lydia Jenner, John Baha Tadros, Steve Milner, James Duncan, Sally Kerr, Louise Nordin, Matt Weston, Olivia Payton, Tofi Oni, Craig Zhao, Sukhdeep Gill, Mohammad Iqbal, Marie-Clare Killen, Khaled Aneiba, Warran Wignadasan, Dilip Pillai, Luke Hughes, Jonathan Crosby, Mike Whitehouse, Thomas Corbett, Arshad Iqbal, Steph Buchan, Laura Beddard, Venkat Vardhan, Becky Beamish, Matt Jones, Jonathan Holley, Rebecca Morrell, Robin Lerner, Kylea Draper

**Affiliations:** 1 Oxford Trauma and Emergency Care, Nuffield Department of Orthopaedics, Rheumatology and Musculoskeletal Sciences, University of Oxford, Trauma Unit, Kadoorie Centre, John Radcliffe Hospital, Oxford, UK; 2 Oxford Clinical Trials Research Unit, Centre for Statistics in Medicine, Nuffield Department of Orthopaedics, Rheumatology and Musculoskeletal Sciences, University of Oxford, Botnar Research Centre, Oxford, UK; 3 Nuffield Department of Primary Care Health Sciences, University of Oxford, Radcliffe Primary Care Building, Oxford, UK; 4 College of Medicine and Health, South Cloisters, University of Exeter, Exeter, UK; 5 Department of Trauma and Orthopaedics, University Hospital Coventry and Warwickshire NHS Trust, University Hospital Coventry and Warwickshire, Coventry, UK; 6 Department of Health Sciences, University of Leicester, George Davies Centre, Leicester, UK

## Abstract

**Objective:**

To assess wrist function, quality of life, and complications in adult patients with a dorsally displaced fracture of the distal radius, treated with either a moulded cast or surgical fixation with K-wires.

**Design:**

Multicentre randomised clinical superiority trial,

**Setting:**

36 hospitals in the UK National Health Service (NHS).

**Participants:**

500 adults aged 16 or over with a dorsally displaced fracture of the distal radius, randomised after manipulation of their fracture (255 to moulded cast; 245 to surgical fixation).

**Interventions:**

Manipulation and moulded cast was compared with manipulation and surgical fixation with K-wires plus cast. Details of the application of the cast and the insertion of the K-wires were at the discretion of the treating surgeon, according to their normal clinical practice.

**Main outcome measures:**

The primary outcome measure was the Patient Rated Wrist Evaluation (PRWE) score at 12 months (five questions about pain and 10 about function and disability; overall score out of 100 (best score=0 and worst score=100)). Secondary outcomes were PRWE score at three and six months, quality of life, and complications, including the need for surgery due to loss of fracture position in the first six weeks.

**Results:**

The mean age of participants was 60 years and 417 (83%) were women; 395 (79%) completed follow-up. No statistically significant difference in the PRWE score was seen at 12 months (cast group (n=200), mean 21.2 (SD 23.1); K-wire group (n=195), mean 20.7 (22.3); adjusted mean difference −0.34 (95% confidence interval −4.33 to 3.66), P=0.87). No difference was seen at earlier time points. In the cast group, 33 (13%) of participants needed surgical fixation for loss of fracture position in the first six weeks compared with one revision surgery in the K-wire group (odds ratio 0.02, 95% confidence interval 0.001 to 0.10).

**Conclusions:**

Among patients with a dorsally displaced distal radius fracture that needed manipulation, surgical fixation with K-wires did not improve patients’ wrist function at 12 months compared with a cast.

**Trial registration:**

ISRCTN registry ISRCTN11980540.

## Introduction

Fractures of the distal radius are extremely common injuries; 6% of women will have sustained such a fracture by the age of 80 and 9% by the age of 90.^1^ However, the optimal management of fractures of the distal radius in adults remains controversial.^1^ ^2^ ^3^ ^4^ ^5^ ^6^ ^7^ In general, if the bone fragments are undisplaced (that is, the bone fragments remain in anatomical alignment), fractures of the distal radius are treated non-operatively. However, if the bone fragments have displaced (moved out of their normal alignment), the treating clinician will usually recommend a “manipulation” of the bone fragments to restore the normal anatomy. Manipulation of a fracture is painful, so this is usually carried out using local, regional, or general anaesthesia.

After the manipulation, the bone fragments can fall back out of normal alignment. Therefore, the bone fragments need to be supported while they heal. This routinely involves surgical implants such as wires, plates, and screws or external fixation. Implants provide reliable fixation of the bone fragments. A moulded cast is an alternative intervention to hold the bone fragments in position. A cast is cheaper than metal implants and avoids the risk of surgical complications, but whether it provides the same functional outcome as surgical fixation is not known.

A recent Cochrane review summarised the existing evidence for surgical fixation with wires for treating distal radial fractures in adults.^2^ All of the trials included in the review were found to be at high risk of bias with incomplete reporting, and the authors were unable to draw a conclusion about the effect of the interventions on patient reported function. The objective of the DRAFFT2 trial was to compare wrist function, quality of life, and complications in patients having a manipulation of a dorsally displaced fracture of the distal radius treated with a moulded cast without metal implants versus surgical fixation with K-wires plus cast.

## Methods

### Study conduct and oversight

The protocol and statistical analysis plan are available online.^8^ ^9^ This multicentre randomised superiority clinical trial was conducted at 36 National Health Service (NHS) hospitals in the UK. The National Research Ethics Committee gave the study a favourable opinion on 6 October 2016, and recruitment centres received permission from their research and development departments. Two minor amendments were made to the protocol to extend the duration of the trial, add digital follow-up, and allow for incentives for follow-up. A local researcher approached eligible patients prospectively and provided them with written information about the trial before asking them to provide informed consent.

### Participants

All patients aged 16 years or over with a dorsally displaced fracture of the distal radius were screened. Patients were potentially eligible if their treating surgeon recommended that they needed a manipulation of their fracture. Patients were excluded if the injury was more than two weeks old, the fracture extended more than 3 cm from radiocarpal joint, the fracture was open with a Gustilo and Anderson grading greater than 1, the joint surface of the fracture could not be reduced by closed manipulation, or the patient was unable to complete follow-up questionnaires. After provision of informed consent, the local research team recorded baseline data, including pre-fracture wrist function (retrospective by recall) and current, post-fracture wrist function.

### Randomisation and blinding

The randomisation sequence, prepared by the trial statistician, included block stratification by recruitment centre, articular extension of the fracture (intra-articular or extra-articular), and age of the participant (<50 or ≥50 years). The treatment allocation was computer generated via a secure, centralised web based randomisation service.

Patients who provided informed consent were advised that at the time of surgery, in a minority of cases, the surgeon might be unable to manipulate the fracture into its normal anatomical alignment without making incisions in the skin to access the fracture site. Patients who needed such an “open” reduction of the articular surface of their fracture were not randomised into the trial and were treated according to their surgeons’ normal clinical practice. For participants in whom a closed reduction of the fracture was possible, the surgeon completed the randomisation process after the manipulation. The participants were allocated in a 1:1 ratio to receive a moulded cast (“cast group”) or surgical fixation with K-wires (“K-wire group”). Surgeons commonly use both techniques in their normal clinical practice. As the interventions were clearly visible to the participants, they could not be blind to treatment allocation.

### Interventions

All surgeons in the UK, and in most parts of the world, are trained in the manipulation of fractures of the distal radius, the insertion of K-wires, and the application of moulded plaster casts; these are very common procedures in orthopaedic trauma (supplementary table D). The manipulation was carried out using local, regional, or general anaesthesia, as per routine clinical practice. An image intensifier x ray machine allowed the surgeon to judge that an adequate closed reduction was achieved after manipulation. In this pragmatic trial, the decision to accept the position after closed reduction was left to the discretion of the treating surgeons, as per their usual practice.

Participants who were randomly allocated to the cast group had a shaped (moulded) cast applied over the skin to hold the bone fragments in position. The cast extended from below the elbow to the metacarpals. In this pragmatic trial, the details of the moulding technique were left to the discretion of the surgeons, as per their usual technique (supplementary table D).

Participants who were randomly allocated to K-wire group had the skin below the elbow prepared with antiseptic and sterile drapes. The K-wires were passed through the skin over the back of the wrist and directly into the bone to hold the bone fragments in the correct position. Different options exist for the positioning of wires. Again, in this pragmatic trial, the size and number of wires, the insertion technique, and the configuration of wires were left to the discretion of the surgeons, as per their normal practice (supplementary table D). A cast was applied at the end of the procedure as per standard practice, but this cast did not need to be specifically moulded as the wires themselves hold the bone in position. We chose K-wire fixation as the surgical intervention comparator because previous research indicates that K-wire fixation provides similar outcomes to the alternative plate and screw fixation for those patients in whom a closed reduction of the fracture can be achieved (plates are reserved for fractures requiring an open reduction to restore the joint surface).^5^

The cast and wires, if inserted, were removed during a clinical follow-up appointment six weeks after treatment. Most patients were discharged at this point; any further clinical follow-up was at the discretion of the treating clinician. Research specific follow-up was completed by electronic/postal questionnaire at three, six, and 12 months.

All patients randomised into the two intervention groups received the same standardised, written physiotherapy advice detailing the exercises they needed to perform for rehabilitation following their injury. Any additional rehabilitation activities beyond those on the written information sheet (including a formal referral to physiotherapy) were at the discretion of the clinical team, the patient, or both.

### Outcome measures

The primary outcome measure was the Patient Rated Wrist Evaluation (PRWE) score at 12 months post-randomisation.^10^ ^11^ The PRWE rates wrist function by using a range of questions in two, equally weighted, subscales concerning the patient’s experience of pain and function. Scoring for all the questions is via a 0-10 scale ranging from “no pain” or “no difficulty” (0) to “worst possible pain” or “unable to do.”^10^ Five questions relate to a patient’s experience of pain and 10 relate to function and disability; scores for the 10 function items are summed and divided by two and added to the five pain items to give a score out of 100 (best score=0 and worst score=100). In line with the outcome measure guidelines,^12^ a question that has not been completed in each of the subscales can be replaced by the mean score of the subscale.

The secondary outcomes were the PRWE score at three and six months post-randomisation and area under the curve for the PRWE over the 12 months using all time points; health related quality of life measured by EQ-5D-5L at three, six, and 12 months^13^; complications including further surgical interventions for loss of reduction, reported during the first year post-randomisation; and cost-effectiveness (reported separately).

### Statistical analysis

A previous trial by our group,^5^ which included the same patient population, showed a normal distribution of the PRWE scores at 12 months with a standard deviation of 16 points. However, other studies showed greater variance,^14^ so we used a conservative estimate of the standard deviation of 18 points for the sample size calculation. We set the minimal clinically important difference at six points. MacDermid et al found that the PRWE is sensitive enough to detect subtle but clinically relevant changes in wrist function of this order of magnitude in patients sustaining a fracture of the distal radius^11^; a mean difference of six points in the PRWE score is just above the amount achieved if all the participants in one group responded that they had one degree better response to any of the PRWE’s constituent question (for example, one degree less difficulty in turning a doorknob) than the other group. For 90% power to detect the minimal clinically important difference at the two sided 5% significance level, we needed a total of 382 participants. A margin of 20% loss of primary outcome data required a minimum of 476 participants to be recruited.

We analysed the primary outcome measure, PRWE score at 12 months post-randomisation, on an intention to treat basis, meaning that all randomised participants were analysed in the intervention groups to which they were randomly allocated, irrespective of the intervention received. If participants had observed data at any of the time points, we included them in the analysis. We used a linear mixed effects model for the primary outcome. This model accounted for person within recruitment centre as a random effect,^15^ and baseline (post-injury) values, other time points, articular extension of the fracture (intra or extra), and age of the patient (<50 years or ≥50) as fixed effects. The model also included treatment by time point interactions to allow time specific treatment effects to be calculated. Time was included as a discrete variable with indicator variables for time points of baseline (post-injury) and three, six, and 12 months. We included baseline as a response, rather than adjusting for it as a continuous covariate, to obtain time point specific estimates for use in the secondary area under the curve model. We used the delta method to calculate the standard errors for these estimates. We also used a linear mixed effects model to analyse the EQ-5D-5L outcomes. We analysed complications by calculating the odds ratio and 95% confidence interval using logistic regression adjusted for the stratification variables in the intention to treat population.

We did multiple supporting analyses on the primary outcome, to examine the robustness of conclusions to different assumptions. One such analysis was on a per protocol population defined as all participants who received the treatment to which they were randomised with data on the primary outcome. We also did subgroup analyses of the two clinical stratifying variables (age and intra-articular extension) on the primary outcome. Details of the assessment of missing data can be found in the statistical analysis plan. We summarised the PRWE across all time points descriptively, comparing participants who did and did not have further surgery due to loss of fracture reduction in the first six weeks (supplementary table C).

All tests were two sided and considered to provide evidence for a significant difference if the P value was 0.05 or less (5% significance level). We used R version 3.6.1 for all analyses.

### Patient and public involvement

The UK Musculoskeletal Trauma Patient and Public Involvement (PPI) Group co-designed this study, with particular regard to the choice of outcome measures and the follow-up arrangements, which were designed to limit the number of face-to-face hospital visits needed. Subsequently, a patient representative from this group became a member of the DRAFFT2 Trial Management Group, overseeing all elements of the set-up and delivery of the trial and the dissemination of the lay summary at completion. Another patient representative was a member of the independent Trial Steering Committee. We will produce a written lay summary of the trial results and an explainer video aimed at patients and the public, to be distributed via the study website, social media, and the Musculoskeletal Trauma PPI group.

## Results

**Fig 1:**
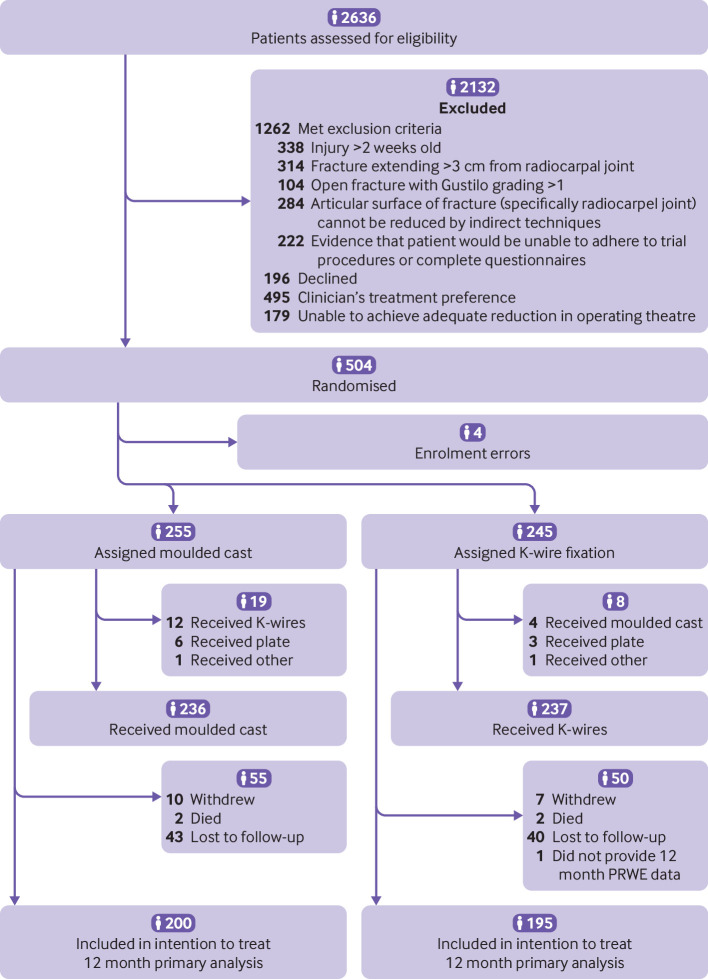
Consort flow diagram

Between January 2017 and March 2019, 500 participants were randomly allocated to receive a cast (n=255) or K-wire fixation (n=245) after manipulation of their displaced fracture. We included 395 (79%) participants in the primary analysis at 12 months. Seventeen participants withdrew (10 in the cast group and seven in the K-wire group) and four died (two in each group) (fig 1).

Participants had a mean age of 60.1 (SD 16.6) years and were mostly female (417; 83%). The intervention groups were well balanced on all baseline characteristics (table 1). Nearly all of the interventions in both groups of the trial were performed by, or under the supervision of, a consultant surgeon.

**Table 1:** Baseline characteristics. Values are numbers (percentages) unless stated otherwise

Characteristics	Cast (n=255)	K-wire (n=245)
Age (discrete):		
<50 years	62 (24)	51 (21)
≥50 years	193 (76)	194 (79)
Mean (SD) age (continuous), years	59.6 (17)	60.7 (16)
Female sex	212 (83)	205 (84)
Articular extension of fracture:		
Extra-articular extension	184 (72)	176 (72)
Intra-articular extension	71 (28)	69 (28)
Treated wrist:		
Left	147 (58)	146 (60)
Right	108 (42)	98 (40)
Not documented	0 (0.0)	1 (0.4)
Baseline outcomes pre-injury (retrospective):		
Mean (SD) PRWE score	3.1 (10.3)	4.5 (14.0)
Mean (SD) EQ-5D-5L index score	0.9 (0.2)	0.9 (0.1)
Mean (SD) EQ-VAS score, 0-100	84.6 (15.3)	86.4 (13.9)
Baseline outcomes post-injury:		
Mean (SD) PRWE score	84.3 (13.3)	81.9 (14.5)
Mean (SD) EQ-5D-5L index score	0.3 (0.3)	0.4 (0.3)
Mean (SD) EQ-VAS score, 0-100	63.9 (22.8)	64.2 (23.1)

EQ-5D-5L=EuroQol five dimension five level scale; EQ-VAS=EuroQol visual analogue scale; PRWE=Patient Rated Wrist Evaluation.

### Primary outcome measure

Participants in both groups showed improvement in their PRWE score during the 12 months after their injury, but they did not achieve their pre-injury level of wrist function. We found no evidence of a difference in the PRWE score at 12 months post-randomisation between the two intervention groups (cast group (n=200), mean 21.16 (SD 23.09); K-wire group (n=195), mean 20.69 (22.33); adjusted mean difference −0.34 (95% confidence interval −4.33 to 3.66), P=0.87) (table 2). Nor did we find evidence of a difference in PRWE score at three or six months post-randomisation or in the area under the curve analysis over the entire 12 month period.

**Table 2:** Patient Reported Wrist Evaluation results in intention to treat population

Time point	Cast			K-wire			Mean difference		P value
	No	Mean (SD)		No	Mean (SD)		Unadjusted	Adjusted (95% CI)	
Baseline (post-injury)	253	84.3 (13.30)		243	81.91 (14.52)		−2.39	-	-
3 months	213	42.08 (23.85)		201	41.56 (24.77)		−0.51	−0.45 (−4.37 to 3.47)	0.82
6 months	202	28.35 (23.35)		206	27.56 (22.33)		−0.79	−0.32 (−4.26 to 3.62)	0.87
12 months (primary outcome)	200	21.16 (23.09)		195	20.69 (22.33)		−0.47	−0.34 (−4.33 to 3.66)	0.87
Area under curve over 12 months	-	38.19*		-	37.60*		-	−0.60 (−4.41 to 3.21)	0.88

* Model estimate. Analysis based on mixed effects model with repeated measures from all time points.

### Secondary outcomes

Health related quality of life measured by the EQ-5D-5L index also showed improvement over time from injury until 12 months, but we observed no difference between the intervention groups at any time point or in the area under the curve analysis, with a 12 month adjusted mean difference of −0.03 (−0.07 to 0.02) (table 3). The EQ-5D visual analogue scale showed similar findings; improvement occurred throughout the follow-up period, but no difference existed between intervention groups at any time point or in the area under the curve analysis, with a 12 month adjusted mean difference of −0.51 (−4.30 to 3.29). See supplement 1 for the full results tables.

**Table 3:** Secondary continuous outcome results in intention to treat population

Time point	Cast			K-wire			Mean difference		P value
	No	Mean (SD)		No	Mean (SD)		Unadjusted	Adjusted (95% CI)	
**EQ-5D-5L index**									
Baseline (post-injury)	252	0.35 (0.28)		241	0.37 (0.26)		0.02	-	-
3 months	217	0.68 (0.21)		203	0.67 (0.20)		−0.01	−0.02 (−0.06 to 0.02)	0.37
6 months	204	0.75 (0.21)		207	0.75 (0.18)		0.00	0.00 (−0.04 to 0.04)	0.96
12 months	199	0.81 (0.20)		197	0.78 (0.21)		−0.02	−0.03 (−0.07 to 0.02)	0.26
Area under curve over 12 months	-	0.69*		-	0.68*		-	−0.01 (−0.05 to 0.03)	0.82
**EQ-VAS**									
Baseline (post-injury)	253	63.87 (22.76)		242	64.19 (23.14)		0.32		-
3 months	216	77.08 (18.25)		201	75.55 (18.51)		−1.53	−1.73 (−5.44 to 1.98)	0.36
6 months	202	79.29 (20.02)		204	81.09 (16.37)		1.80	1.87 (−1.88 to 5.61)	0.33
12 months	199	81.11 (18.06)		195	80.42 (18.00)		−0.69	−0.51 (−4.30 to 3.29)	0.79
Area under curve over 12 months		76.81*		-	76.98*		-	0.16 (−3.59 to 3.92)	0.97

EQ-5D-5L=EuroQol five dimension five level scale; EQ-VAS=EuroQol Visual Analogue Scale.

* Model estimate. Analysis based on mixed effects model with repeated measures from all time points.

Thirty three (13%) participants in the cast group had surgery for loss of fracture reduction in the first six weeks post-randomisation versus one (0.4%) in the K-wire group (odds ratio 0.02, 95% confidence interval 0.001 to 0.10; P<0.001) (table 4). The PRWE scores across all time points for participants in the cast group who needed further surgery owing to loss of reduction in the first six weeks were very similar to those for the cast group as a whole (supplementary table C). This could imply that participants who received further surgery owing to loss of reduction experienced no worse wrist function than those who did not have further surgery. However, as we did no formal statistical evaluation, the groups may have underlying differences, and the number of events was low, so these data should be interpreted with caution. Other complications were rare, with no evidence of a difference between the two intervention groups (28 in the cast group, 22 in the K-wire group); these complications included blood clots and complex regional pain syndrome.

**Table 4:** Complications. Values are numbers (percentages) unless stated otherwise

Complications	Cast (n=255)	K-wire (n=245)	Odds ratio (95% CI)	P value
Loss of fracture reduction at 6 weeks	33 (13)	1 (0.4)	0.02 (0.001 to 0.10)	<0.001
Further surgery up to 12 months*	37 (15)	5 (2)	0.08 (0.02 to 0.20)	<0.001
Complex regional pain syndrome	5 (2)	7 (3)	1.50 (0.43 to 5.28)	0.55
Deep venous thrombosis or pulmonary embolus	1 (0.4)	1 (0.4)	0.75 (0.03 to 22.58)	0.85

* Other reasons for further surgery that were not due to loss of fracture reduction included subsequent unrelated fall, carpel tunnel, tendon transfer, and stiffness.

All supporting analyses showed no significant difference between intervention groups at any time point for the PRWE score. The 12 month adjusted mean differences were −0.80 (−5.11 to 3.52) in the per protocol analysis (supplementary table A), 0.65 (−3.70 to 4.99) in the three level model (including surgeon effect; supplementary table B), and no significant difference for the subgroups based on age or intra-articular extension of the fracture (supplementary figure A). Analysis showed that the conclusions were robust to missing data assumptions.

## Discussion

This randomised clinical trial found no evidence that manipulation and surgical fixation with K-wires was superior to manipulation and a moulded cast, in terms of pain and function at 12 months, in the management of dorsally displaced distal radius fractures. Nor did it find evidence of a difference between the interventions at three or six months or in the total area under the curve for the PRWE score. Both intervention groups showed improvement over the 12 months period, but participants did not reach pre-injury levels of wrist function. Health related quality of life showed a similar pattern of recovery over time, and again the difference in EQ-5D-5L utility score was not significant.

We found a statistically significant difference in the number of further interventions needed for loss of fracture reduction in the six weeks after the manipulation of the fracture. In this trial, the decision to offer participants further surgery for loss of position of the fracture was left to the discretion of the treating clinicians as per their routine clinical practice. Surgeons’ thresholds for offering further surgery may vary, but the lower rate of further surgery in the K-wire group was not unexpected as the purpose of surgical fixation is to secure the reduction of the fracture by using metalwork—in this case, by passing metal wires across the fracture to hold the bone fragments in their anatomical position while they heal. By contrast, a moulded cast provides only indirect support to the bones after their manipulation. As soft tissue swelling settles after the injury, the cast becomes looser and the reduction of the bone fragments may be lost. These data provide important information for clinicians advising patients about their treatment options following this common injury and indicate that patients should be followed up carefully for signs of loss of fracture reduction. If treated with a moulded cast, 87% of patients will not need a surgical fixation but 13% (one in eight) will need a further intervention for loss of fracture reduction in the first six weeks.

### Comparison with previous studies

We chose K-wire fixation as the surgical intervention comparator, as previous research indicates that K-wire fixation provides similar outcomes to the alternative plate and screw fixation for those patients in whom a closed reduction of the fracture can be achieved, with plates being reserved for fractures requiring an open reduction of the articular surface of the fracture.^5^ The use of wires reflects current UK National Institute for Health and Care Excellence guidelines for the management of distal radius fractures.^7^ After the DRAFFT2 trial started, Karantana et al published a Cochrane review of wire fixation for adult patients with a dorsally displaced fracture of the distal radius.^2^ This review included a total of 11 randomised or quasi-randomised trials of manipulation and plaster cast versus manipulation and K-wire fixation, involving 917 participants nearly all of whom were older adults. The authors concluded that the quality of the existing evidence was very low. All of the trials included in the review were found to be at high risk of bias, with incomplete reporting meaning that the authors were unable to draw a conclusion about the effect of the interventions on patient reported function. They did report that the risk of re-displacement of the fracture in the participants treated with manipulation and cast was very similar to that found in DRAFFT2, with an average of 12% (range 3-75%). We updated this evidence synthesis in July 2020 with a search of the NIH National Library of Medicine via PubMed.gov and did not find any other trials that attempted to answer this question.

### Strengths and limitations of study

The strengths of DRAFFT2 were the use of multiple centres and clinicians reflecting the care provided across a healthcare system; the large number of participants, including adults of all ages; and the use of validated patient reported outcomes.

Recruiting patients to trials in the context of urgent surgery is difficult. A concern before this trial started was that patients, surgeons, or both would not be willing to take part. In fact, only 196 patients declined to take part in the study. A bigger limitation was equipoise within the surgical community. Four hundred and ninety five potentially eligible patients were not offered the chance to take part in the trial because the treating surgeon had a clinical preference for a particular treatment; 120 patients were offered K-wire fixation, 31 manipulation and cast, and 344 other treatments or unspecified. Although these numbers are low in relation to the total of 2636 patients who were screened as part of the study, they may affect the external validity of the trial. A further anticipated limitation was crossover of participants from the allocated intervention. However, only 27 of the 500 participants did not receive their allocated intervention. The secondary per protocol analysis of participants who received their allocated intervention confirmed the results of the primary intention to treat analysis, finding no evidence of a difference between the groups.

Loss to follow-up in a trial can reduce the generalisability of the results. Seventy nine per cent of patients in this trial provided primary outcome data, and this was in keeping with the loss to follow-up of 20% planned for in the sample size calculation, which indicated a minimum of 460 participants be recruited. As the trial recruited 500 participants, we can be confident that this clinical trial had the intended level of statistical precision, and this is evidenced by the relatively narrow confidence intervals.

The final major limitation of the trial was that neither the treating clinicians nor the participants could be blind to the interventions. This is inevitable for surgical interventions in which the treatment is clearly visible to the patients. Assessment bias was minimised by the fact that the surgeons providing the interventions played no part in the assessment of the participants’ outcomes.

### Conclusions and policy implications

Surgical fixation with K-wires did not provide better wrist function at 12 months compared with a moulded cast, indicating that a cast is an acceptable first line treatment following manipulation of a dorsally displaced fracture of the distal radius. Cast treatment avoids the expense and risks of surgical fixation for seven out of eight patients. However, careful follow-up is needed as one in eight patients treated with a cast required subsequent surgical intervention as the fracture reduction could not be maintained.

## What is already known on this topic

Surgical fixation provides reliable functional outcomes for patients after manipulation of a displaced fracture of the distal radius, but surgery carries risk for the patient and is expensive
A moulded plaster cast is a safer and cheaper intervention but may not provide the same functional outcome
Which of these treatments is superior is not known

## What this study adds

Surgical fixation with K-wires did not improve patients’ wrist function at one year compared with casting following manipulation of a fracture of the distal radius
However, one in eight patients treated with a moulded cast needed later surgery for loss of fracture position in the first six weeks after their injury

## Data Availability

All data requests should be submitted to the corresponding author for consideration. Access to anonymised data may be granted following review.

## Web extra

Extra material supplied by authors

Web appendix: Supplementary materials

## Notes

Contributors: MLC, JA, SEL, JD, and HH obtained grant funding for this project. MLC, JA, SEL, JD, and HH were responsible for the design and concept of this project and the oversight of the study. AO, MEP, and JAC were responsible for the analysis and interpretation of the study data. All authors were responsible for the writing and revision of the manuscript. All authors reviewed and agreed the final manuscript. MLC and JAC are the guarantors. The corresponding author attests that all listed authors meet authorship criteria and that no others meeting the criteria have been omitted.

The lead authors (the manuscript’s guarantors) affirm that the manuscript is an honest, accurate, and transparent account of the study being reported; that no important aspects of the study have been omitted; and that any discrepancies from the study as planned (and, if relevant, registered) have been explained.

Provenance and peer review: Not commissioned; externally peer reviewed.

Dissemination to participants and related patient and public communities: A lay summary and an animated video will be made available to the study participants and the general public on the study web pages. The animated video and an infographic will also be shared on social media.
