# Supplementary material for: Surgical fixation with K-wires versus casting in adults with fracture of distal radius: DRAFFT2 multicentre randomised clinical trial
Source: BMJ. 2022 Jan 19;376:e068041. doi: 10.1136/bmj-2021-068041 (PMC8767805; doi:10.1136/bmj-2021-068041)
Supplement: Supplementary file 1 — Web appendix: Supplementary materials [file cosm068041.ww1.pdf]

Surgical fixation with K-wires versus casting in adults with fracture of distal radius: DRAFFT2 multicentre randomized clinical trial

Matthew L Costa, PhD (0000-0003-3644-1388), Juul Achten PhD, Alexander Ooms MSc, May Ee Png PhD, Jonathan A Cook PhD, Sarah E Lamb PhD, Helen Hedley PhD, Joseph Dias PhD and DRAFFT2 collaborators

Supplementary data

The supporting analyses presented in Tables A and B reaffirm the conclusions drawn from the primary results that there is no difference between the treatment groups PRWE at any point during the 12 months post-injury. The results in Table B, using a model that allows for possible heterogeneity due to treating surgeon, in comparison with the primary analysis shows there is little difference in results caused by the surgeon performing the procedure. This confirms intuition as both procedures are performed regularly by all surgeons.

Table C shows the PRWE across all time points for participants in the Cast group who had further surgery due to loss of reduction in the first six weeks compared to the Cast group who did not experience any loss of reduction within the first six weeks. The PRWE scores were very similar for these two group. This could imply that those who had further surgery due to loss of reduction experience no worse wrist function post-injury than those who do not have further surgery. As there has been no formal statistical testing performed and the number of events was low, this should be interpreted with caution.

Information regarding operation details is supplied in Table D.

There was no evidence of a differential effect by pre-defined clinical subgroups of interest (age and intra-articular extension status). Treatments by subgroup interactions did not indicate any difference for age (mean difference 3.04, 95% CI (-4.41, 10.49), p-value=0.42) and intra-articular extension status (mean difference -3.16, 95% CI (-9.86, 3.54), p-value=0.35). Figure A shows that whilst there may be small numerical differences between the pre-defined clinical subgroups in this exploratory analysis, any difference is not statistically significant and within the minimum clinically important difference.

Table A PRWE Results in the Per-Protocol Population

	Cast		K-wire		Mean difference		p-value
**Time Point**	**n**	**Mean (SD)**	**n**	**Mean (SD)**	**Unadjusted**	**Adjusted (95% CI)**	
Baseline (post-injury)	188	83.94 (13.62)	189	81.51 (13.67)	-2.43		
3 months	179	42.11 (23.78)	176	40.23 (24.75)	-1.88	-2.12 (-6.51,2.27)	0.34
6 months	178	28.52 (23.16)	186	27.53 (22.51)	-0.99	-1.03 (-5.39,3.32)	0.64
12 months	189	21.73 (23.38)	191	20.85 (22.42)	-0.88	-0.80 (-5.11,3.52)	0.72
Area under the curve over 12 months		38.07*		36.67*		-1.39 (-6.12,3.34)	0.77

* Model estimate. The analysis was based on a mixed effects model with repeated measures from all timepoints. PRWE = Patient Reported Wrist Evaluation.

Table B PRWE Results in the Intention-to-Treat Population Using a Three-Level Model (Including Surgeon Effects)

	Cast		K-wire		Mean difference		p-value
**Time Point**	**n**	**Mean (SD)**	**n**	**Mean (SD)**	**Unadjusted**	**Adjusted (95% CI)**	
Baseline (post-injury)	253	84.3 (13.30)	243	81.91 (14.52)	-2.39		
3 months	213	42.08 (23.85)	201	41.56 (24.77)	-0.51	0.55 (-3.70,4.79)	0.80
6 months	202	28.35 (23.35)	206	27.56 (22.33)	-0.79	0.85 (-3.41,5.12)	0.70
12 months	200	21.16 (23.09)	195	20.69 (22.33)	-0.47	0.65 (-3.70,4.99)	0.77

Table C PRWE Results in the Cast Group with no Loss of Reduction at 6 Weeks Compared

	No Lost Reduction Cast		Lost Reduction Cast	
**Time Point**	**n**	**Mean (SD)**	**n**	**Mean (SD)**
Baseline (post-injury)	220	83.97 (13.77)	33	86.52 (9.51)
3 months	181	41.48 (24.09)	32	45.46 (22.46)
6 months	172	28.21 (23.54)	30	29.17 (22.61)
12 months	172	21.22 (23.47)	28	20.79 (20.95)

(Descriptively) to the Cast Group with Loss of Reduction at 6 Weeks

Table D Operation Details by Treatment Intervention

	Cast (n = 255) n (%)	K-wire (n = 245) n (%)
**Perioperative antibiotic cover used**		
Yes	48 (19.0)	181 (74.8)
No	204 (81.0)	61 (25.2)
**Number of prior patients with a displaced distal radius fracture treated by surgeon**		
0	1 (0.4)	5 (2.0)
<5	4 (1.6)	13 (5.3)
5-10	11 (4.3)	7 (2.9)
11-20	17 (6.7)	18 (7.4)
>20	222 (87.1)	201 (82.4)
**K-Wire Information**		
Number of Wires Used		
1	0 (0.0)	5 (2.0)
2	4 (1.6)	154 (63.1)
3	7 (2.7)	74 (30.3)
>3	1 (0.4)	3 (1.2)
N/A	243 (95.3)	8 (3.3)
Wire Size (mm)		
1.1	0 (0.0)	1 (0.4)
1.6	9 (3.5)	172 (72.6)
Other	3 (1.2)	56 (23.6)
N/A	243 (95.3)	8 (3.4)
Pinning Technique Used		
Interfragmentary	6 (2.4)	91 (38.2)
Kapanji	4 (1.6)	93 (39.1)
Mixed Technique	2 (0.8)	46 (19.3)
N/A	243 (95.3)	8 (3.4)
**Cast information[a]**		
Thumb Included		
Yes	59 (23.2)	
No	176 (69.3)	
N/A	19 (7.5)	
Material Used		
Fiberglass	13 (5.1)	
Plaster of Paris	222 (87.1)	
Other	1 (0.4)	
N/A	19 (7.5)	
Coverage		
Backslab	19 (7.5)	
Full Cast	214 (84.9)	
N/A	19 (7.5)	

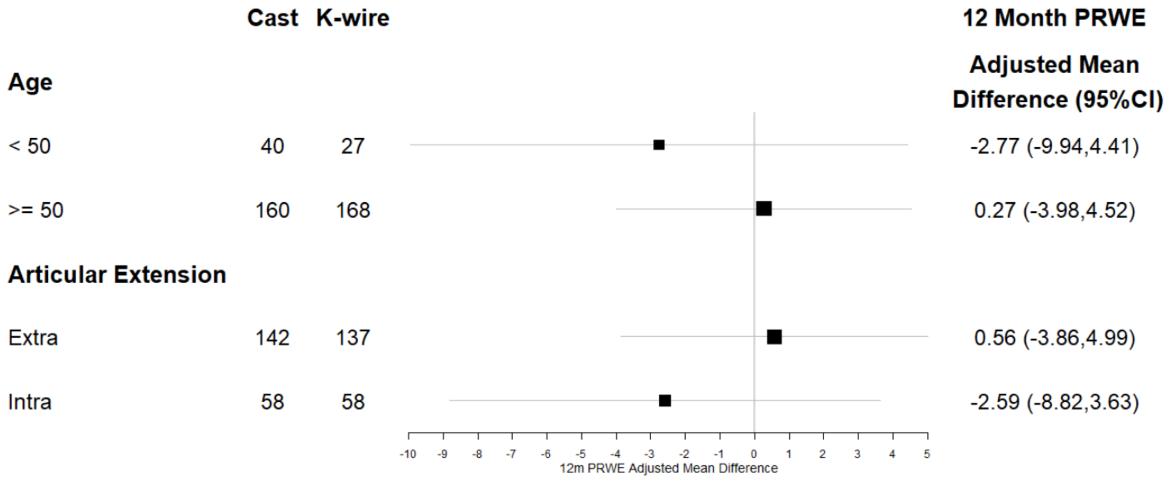

Figure A: 12 Month PRWE Results For Clinical Subgroups. Negative Values imply K-wires are Superior, Positive Values imply Cast is Superior
